# Exploring the Villalta scale to capture postthrombotic syndrome using alternative approaches: A subanalysis of the ATTRACT trial

**DOI:** 10.1016/j.rpth.2022.100032

**Published:** 2022-12-27

**Authors:** Cristina T. Pop, Chu-Shu Gu, Suresh Vedantham, Jean-Philippe Galanaud, Susan R. Kahn

**Affiliations:** 1 Department of Medicine, McGill University, Montreal, Quebec, Canada; 2 Centre for Regulatory Excellence, Statistics and Trials, Ottawa, Ontario, Canada; 3 Washington University in St. Louis, Missouri, USA; 4 University of Toronto, Ontario, Canada; 5 Centre of Excellence in Thrombosis and Anticoagulation Care, Centre for Clinical Epidemiology of the Lady Davis Institute for Medical Research, Montreal, Quebec, Canada

**Keywords:** deep vein thrombosis, postthrombotic syndrome, quality of life, diagnosis, Villalta scale

## Abstract

**Background:**

Clinical trials that evaluated interventions to prevent postthrombotic syndrome (PTS) used the Villalta scale (VS) to define PTS, but there is a lack of consistency in its use.

**Objectives:**

This study aimed to improve the ability to identify patients with clinically meaningful PTS after DVT in participants of the ATTRACT trial.

**Methods:**

We conducted a post hoc exploratory analysis of 691 patients from the ATTRACT study, a randomized trial evaluating the effectiveness of pharmacomechanical thrombolysis to prevent PTS in proximal deep vein thrombosis. We compared 8 VS approaches to classify patients with or without PTS in terms of their ability to discriminate between those with poorer vs better venous disease-specific quality of life (Venous Insufficiency Epidemiological and Economic Study Quality of Life [VEINES-QOL]) between 6- and 24-months follow-up. The difference in the average area under the fitted curve of VEINES-QOL scores between PTS and no PTS ($\Delta \bar{AUC}$) were compared among approaches.

**Results:**

For any PTS (a single VS score ≥5), approaches 1 to 3 had similar $\Delta \bar{AUC}$ (−21.2, −23.7, −22.0, respectively). Adjusting the VS for contralateral chronic venous insufficiency (CVI) or restricting to patients without baseline CVI (approaches 7 and 8) did not improve $\Delta \bar{AUC}$ (−13.6, −19.9, respectively; *P* >.01). For moderate-to-severe PTS (a single VS score ≥10), approaches 5 and 6 requiring 2 positive assessments had greater but not statistically significant $\Delta \bar{AUC}$ than approach 4, using one single positive assessment (−31.7, −31.0, −25.5, respectively; *P* >.01).

**Conclusion:**

A single VS score of ≥ 5 reliably distinguishes patients with clinically meaningful PTS as assessed by impact on QOL and is preferred because of greater convenience (only one assessment needed). Alternative methods to define PTS (ie, adjusting for CVI) do not improve the scale’s ability to identify clinically meaningful PTS.

## Essentials

• The Villalta scale (VS) is used to define postthrombotic syndrome (PTS), often inconsistently.
• We compared 8 different VS approaches to identify PTS as assessed by impact on quality of life.
• A single VS score of ≥ 5 reliably distinguishes patients who have clinically meaningful PTS.
• Alternative methods do not improve the ability to identify PTS that impacts quality of life.

## 1 Introduction

The postthrombotic syndrome (PTS) refers to a clinical condition of chronic venous insufficiency (CVI) that is estimated to develop in 20% to 50% of patients after deep venous thrombosis (DVT) despite optimal anticoagulation therapy [1]. Furthermore, PTS is a common complication of DVT and constitutes a significant burden because it impairs the quality of life (QOL), causes major functional disability and is associated with considerable medical costs [[2], [3], [4]]. PTS is characterized by a wide range of physical signs and symptoms, such as limb pain, swelling, dilation of superficial veins, stasis dermatitis, and leg ulceration [5].

There is no objective diagnostic test to define PTS and, as such, diagnosis relies heavily on a set of subjective symptoms and signs. Different scoring systems have been proposed in the past, making it difficult to standardize the definition of PTS [[6], [7], [8], [9]]. In 2009, the subcommittee on Control of Anticoagulation of the Scientific and Standardization Committee of the International Society on Thrombosis and Hemostasis (ISTH) recommended the use of the Villalta scale (VS) as a standard in the clinical setting to diagnose PTS because of its practicality, good interrater reliability, and external validity [10,11].

Recent trials evaluating interventions to prevent PTS, such as the Compression Stockings to Prevent the Post-Thrombotic Syndrome (SOX) and the Acute Venous Thrombosis: Thrombus Removal with Adjunctive Catheter-Directed Thrombolysis (ATTRACT) trial used the VS to denote PTS, but there is a lack of consistency in its use [12,13]. Several prospective studies and trials have used the original VS scoring method by Prandoni et al. [14,15] comprising 2 consecutive scores ≥5 with an interval of ≥3 months apart as opposed to the ISTH definition of one score ≥5 [16]. Such differences in the definition of PTS makes comparison of research results challenging. The VS is also limited by its low specificity for PTS, as opposed to other forms of chronic venous disease and other diseases affecting the lower limb. A subanalysis of the REcurrent VEnous thromboembolism Risk Stratification Evaluation (REVERSE) study observed that 40% to 50% of diagnosed PTS may partly reflect primary CVI when the leg contralateral to DVT is also scored high (>4) on the VS [17]. Because it is not possible to document the VS score before DVT occurrence in research subjects enrolled into studies based on having acute DVT, abnormal VS scores could be explained wholly or partly by CVI predating DVT, rather than by PTS that developed after DVT.

The ATTRACT trial compared the effectiveness of pharmacomechanical catheter-directed thrombolysis (PCDT) to no PCDT to prevent PTS in patients with proximal DVT. The primary study end point, PTS, was defined by the VS using a cut-off of ≥5 [13]. In this post hoc exploratory analysis of the ATTRACT trial, we compared 8 different approaches of using the VS to define PTS in ATTRACT trial participants, with the primary objective of improving the ability to identify patients who develop clinically meaningful PTS after DVT, as reflected by poorer venous disease-specific QOL during the 24-month follow-up.

## 2 Methods

The ATTRACT trial was a phase 3, multicenter, open label, randomized controlled trial sponsored by the National Heart, Lung, and Blood Institute of the National Institutes of Health. Eligible patients with symptomatic proximal DVT affecting the femoral, common femoral, or iliac veins were assigned to receive PCDT and standard treatment or standard treatment alone as per published guidelines with initial and long-term anticoagulation, without procedural intervention, defined as no PCDT. All patients received sized-to-fit, knee-high, 30 to 40-mm Hg elastic compression stockings [13,18]. Patients aged <16 or >75 years, were pregnant, had symptoms for >14 days, had preexisting PTS, or previously diagnosed ipsilateral DVT 2 years earlier were excluded. Patients were assessed at baseline, day 10, and 1, 6, 12, 18, and 24 months after randomization. The study’s primary outcome was the development of PTS, defined as a VS score of ≥5 or an ulcer in the leg with the index DVT, at any time between the 6-month and 24-month follow-up visits. Patients with a venous leg ulcer in the index leg were classified as having moderate-to-severe PTS, irrespective of the summed VS score [18]. As well, patients who underwent an unplanned endovascular procedure to treat severe symptoms beyond 6 months after randomization were also counted as having “any” PTS [18]. The VS is graded on the presence and severity of 5 symptoms and 6 clinical signs of the lower limb (each rated on severity from 0 to 3 points), and on the presence or absence of a venous ulcer. The total VS score, ranging from 0 to 33, was assessed in both legs and used to categorize the severity of PTS in the index leg as mild (score, 5-9), moderate (score, 10-14), or severe (score, ≥15 or presence of ulceration) at each follow-up visit.

Validated, patient-reported assessment tools were used at baseline and during all follow-up visits to measure the venous disease-specific QOL using the Venous Insufficiency Epidemiological and Economic Study Quality of Life (VEINES-QOL) [7,19,20]. The VEINES-QOL is a disease-specific QOL instrument that has been validated for chronic venous diseases of the leg, DVT, and venous leg ulcers [21]. The original study has been replicated in 3 other validity studies of mixed cohorts [[22], [23], [24]]. It has been shown to be an acceptable tool owing to its reliability, internal and external validity, and responsiveness for use as a patient-reported measure of outcome in DVT [25]. The VEINES-QOL instrument relies on 25 items to form a summary score. Higher scores reflect better QOL.

The trial was approved by the local ethics committees, and all participants provided written informed consent. Full eligibility criteria, study design, and detailed description of the trial methods are provided in the primary publication [18].

### 2.1 Approaches to defining PTS for this analysis

For this analysis, we explored 8 different approaches, based on the VS, to classify patients as having or not having PTS during the 24 months of follow-up: 1) Ipsilateral-VS (Ipsi-VS) score of ≥5 at least once from 6 to 24 months follow-up (ISTH definition); 2) Ipsi-VS score of ≥5 on ≥2 consecutive assessments from 6 to 24 months follow-up (Prandoni definition); 3) Ipsi-VS score of ≥5 on any ≥2 assessments (not necessarily consecutive) from 6 to 24 months follow-up; 4) Ipsi-VS score of ≥10 at least once from 6 to 24 months follow-up (ISTH definition for moderate-to-severe PTS); 5) Ipsi-VS score of ≥10 on ≥2 consecutive assessments from 6 to 24 months follow-up (Prandoni definition for moderate-to-severe PTS); 6) Ipsi-VS score of ≥10 on any ≥2 assessments (not necessarily consecutive) from 6 to 24 months follow-up; 7) Ipsi-VS score minus contralateral VS (contra-VS) score of ≥5 at least once from 6 to 24 months follow-up; and 8) Ipsi-VS score of ≥5 at least once from 6 to 24 months follow-up, but only assessed in (ie, restricted to) patients who had a VS score of <5 in the contralateral leg at baseline. For each approach, the proportion of patients who met criteria for PTS was calculated in a binary form (ie, yes/no), based on the criteria for each definition. Patients with VS of ≥10 on ≥1 assessment were considered to have “any” PTS as well as “moderate-to-severe” PTS. These approaches and the constructs they represent are shown in Table 1.

**Table 1:** Approaches used to define PTS.

Approach	Definition	Construct
1	Ipsilateral-VS score of ≥5 at least once from 6-24 mos follow-up^a^	PTS
2	Ipsilateral-VS ≥5 on 2 or more consecutive assessments from 6-24 mos follow-up	PTS
3	Ipsilateral-VS ≥5 on any 2 or more assessments (not necessarily consecutive) from 6-24 mos follow-up	PTS
4	Ipsilateral-VS score of ≥10 at least once from 6-24 mos follow-up^b^	Moderate-to-severe PTS
5	Ipsilateral-VS ≥10 on 2 or more consecutive assessments from 6-24 mos follow-up	Moderate-to-severe PTS
6	Ipsilateral-VS ≥10 on any 2 or more assessments (not necessarily consecutive) from 6-24 mos follow-up	Moderate-to-severe PTS
7	Ipsilateral-VS minus contralateral VS score of ≥5 at least once from 6-24 mos follow-up	PTS, adjusted for contralateral CVI
8	Ipsilateral-VS score of ≥5 at least once from 6-24 mos follow-up, only assessed in patients with VS score of <5 in the contralateral leg at baseline	PTS, only assessed in patients without baseline CVI

CVI, chronic venous insufficiency; PTS, postthrombotic syndrome; VS, Villalta scale.

a ATTRACT trial primary outcome.

b ATTRACT trial secondary outcome.

### 2.2 Statistical analysis

The primary aim of the analysis was to compare 8 different approaches to using the VS to classify patients as having or not having PTS in terms of their ability to discriminate between patients with DVT with poorer or better VEINES-QOL scores over 24 months follow-up. For each approach, the proportion of ATTRACT patients classified as having PTS, moderate-to-severe PTS and not having PTS was calculated, and the average area under the fitted curve ($\bar{AUC}$) of VEINES-QOL scores between the 6-month and 24-month visits was compared for patients with and without PTS, as an indicator of how well a given approach is able to separate patients into those with better or worse venous disease-specific QOL.

Group means and SEs of the VEINES-QOL and VS scores were calculated at 6- and 24- months follow-up for each of the 8 approaches defining PTS. The VEINES-QOL score trajectory was then estimated for patients classified with and without PTS (ie, group variable) using the growth curve mixed model with piecewise linear regression. The model considers the correlation between the repeated observations and is adjusted by independent clinically important covariates (eg, age, body mass index, baseline VS score, sex, treatment allocation [ie, PCDT vs no PCDT], and extent of DVT). The VEINES-QOL scores were measured at months 6, 12, 18, and 24; these time points were used in piecewise linear regression accordingly, which are represented by an intercept and multiple slopes for each group (eg, PTS and no PTS) in the model. For the residuals, unstructured correlation structure was used.

The difference in VEINES-QOL scores, expressed as delta (Δ) $\bar{AUC}$, was calculated for each approach by subtracting the $\bar{AUC}$ of the VEINES-QOL score from 6 to 24 months for the PTS group by the $\bar{AUC}$ for the non-PTS group. The $\bar{AUC}$ is calculated by the AUC divided by the time period (ie, 18 months), representing the average VEINES-QOL score from 6 to 24 months. The differences of $\Delta \bar{AUC}$, calculated by subtracting the $\Delta \bar{AUC}$ from one PTS definition to another, were compared among the 8 approaches to determine which approach appeared to be the most discriminatory in identifying patients with clinically relevant impaired QOL. Clinically meaningful PTS refers to PTS that has a measurable impact on QOL, operationalized in this study as a definition of PTS that is associated with poorer (or lower) VEINES-QOL score between the 6- and 24-months follow-up, as assessed by a larger $\Delta \bar{AUC}$. This was performed under the assumption that an improved, more specific definition of PTS, will result in a better separation of 2 compared curves. The bootstrap method was used to test if there was a significant difference between 2 $\Delta \bar{AUC}\text{.}$

A two-sided *P* value of ≤.01 was considered to indicate statistical significance for all analyses. Statistical analyses were performed using SAS software version 9.4.

## 3 Results

From December 2009 to December 2014, 692 patients were randomized in the ATTRACT trial (PCDT = 337; no PCDT = 355). One patient in the PCDT group was excluded from all analyses because of ineligibility, leaving 691 patients in the modified intention-to-treat analysis. Baseline characteristics of the patient population were similar between intervention groups (Table 2). In the study cohort, during the 24-months follow-up, 500 of 691 patients completed follow-up; among 191 patients who did not complete follow-up, 15 patients died, 28 withdrew consent, and 148 were lost to follow-up. Regarding PTS assessments, 415 patients completed 4 PTS assessments, 196 patients completed 1 to 3 PTS assessments, and 80 patients missed all 4 PTS assessments [13]. Of the 679 subjects who had a baseline QOL questionnaire, 478 (70%) patients had a complete questionnaire at 24-months, and only 12 of 691 (2%) patients did not fill a QOL questionnaire at baseline.

**Table 2:** Baseline characteristics.

Characteristics^a^	PCDT (N = 336)	No PCDT (N = 355)	Total (N = 691)
Age, y; mean (SD)	51 (14)	51 (13)	51 (13)
Male sex, no. (%)	205 (61)	221 (62)	426 (62)
Race, no. (%)			
White	265 (79)	276 (78)	541 (78)
Black/African American	61 (18)	62 (17)	123 (18)
Other^b^	10 (3)	17 (5)	27 (4)
Weight, kg; mean (SD)	97 (25)	96 (24)	97 (24)
BMI, kg/m^2^; mean (SD)	32 (7.5)	31 (7.7)	32 (7.6)
DVT risk factors, no. (%)			
Major surgery	27 (8)	34 (10)	61 (9)
Hospitalization	26 (8)	38 (11)	64 (9)
Plaster cast immobilization	8 (2)	9 (3)	17 (2)
Childbirth	3 (1)	5 (1)	8 (1)
DVT symptom duration before randomization–days; mean (SD)	6.8 (4.1)	7.0 (4.4)	6.9 (4.2)
Side of Index DVT, no. (%)			
Left	207 (62)	218 (61)	425 (62)
Right	129 (38)	137 (39)	266 (38)
DVT in the common femoral ± iliac vein, no. (%)	195 (58)	196 (55)	391 (57)
Previous DVT or PE or both, no. (%)			
No	253 (75)	268 (75)	521 (75)
Yes^c^	83 (25)	87 (25)	170 (25)
Any previous DVT^d^–no. (%)	75/83 (90)	84/87 (97)	159/170 (94)
Any previous PE^d^–no. (%)	21/83 (25)	16/87 (18)	37/170 (22)
Previous DVT^e^–no. (%)			
Ipsilateral leg	3/75 (4)	13/84 (15)	16/159 (10)
Contralateral leg	37/75 (49)	40/84 (48)	77/159 (48)

BMI, body mass index; DVT, deep vein thrombosis; PCDT, pharmacomechanical catheter-directed thrombolysis; PE, pulmonary embolism.

a Baseline characteristics and table details were reproduced from the original ATTRACT trial [13] article.

b Other includes Asian, American Indian/Alaska Native, Native Hawaiian/Other Pacific Islander, and not reported or refused to answer.

c Subjects may fit into more than one category.

d Denominators for “any previous DVT” and “any previous PE” are obtained from the total number of “previous DVT or PE or both,” for PCDT, no PCDT, and total, respectively.

e Denominators for ipsilateral and contralateral leg of “previous DVT” are obtained from the total number of “any previous DVT,” for PCDT, no PCDT, and total, respectively.

Using approach 1, the ISTH definition of PTS used for the ATTRACT trial’s primary outcome, PTS occurred in 328 patients of 691 (47%) [13]. For PTS defined as a VS score ≥5 on 2 assessments, the Prandoni definition, 23% (159/691) of patients met criteria for PTS (approach 2), and 29% (200/691) of patients met criteria for PTS using approach 3. Using approach 4, the ISTH definition of moderate-to-severe PTS and a secondary outcome in the ATTRACT trial, moderate-to-severe PTS occurred in 21% (144/691) of patients [13]. For moderate-to-severe PTS defined as a VS score ≥10 on 2 assessments, the Prandoni definition, 8% (59/691) of patients met criteria for PTS, and 11% (73/691) of patients met criteria using approach 6. Using approach 7, ie, adjusting the ipsilateral-VS score for the contralateral leg’s score, PTS occurred in 27% (189/691) of the patients. Finally, using approach 8, ie, restricting to patients without CVI at the baseline visit, PTS occurred in 44% (265/607) of patients (Table 3).

**Table 3:** Differences of average area under the fitted curve ($\Delta \bar{AUC}$) between patients categorized as having PTS vs not having PTS using the 8 different approaches to defining PTS.

Approach	n/N (%)	$\Delta \bar{AUC}$^a^ (SE) PTS − No PTS	95% **confidence interval**^b^
1	328/691 (47)	−21.2 (1.5)	−24.2, −18.3
2	159/691 (23)	−23.7 (1.7)	−27.0, −20.5
3	200/691 (29)	−22.0 (1.6)	−25.2, −18.9
4	144/691 (21)	−25.5 (1.8)	−28.9, −22.0
5	58/691 (8)	−31.7 (2.6)	−36.7, −26.6
6	73/691 (11)	−31.0 (2.3)	−35.4, −26.6
7	189/691 (27)	−13.6 (1.8)	−17.1, −10.2
8	265/607 (44)	−19.9 (1.5)	−22.8, −17.1

AUC, area under curve; PTS, Postthrombotic syndrome; (delta), change.

a Differences of average area under the fitted curve ($\Delta \bar{AUC}$) calculated as the difference in average area under the fitted curve in VEINES-QOL scores between 6-months and 24-months in patients with and without PTS using growth curve models with piecewise linear regression adjusting for the extent of deep vein thrombosis and clinical center and for baseline covariates (age, sex, body mass index, and Villalta score).

b Obtained by the bootstrap method.

**Figure 1:**
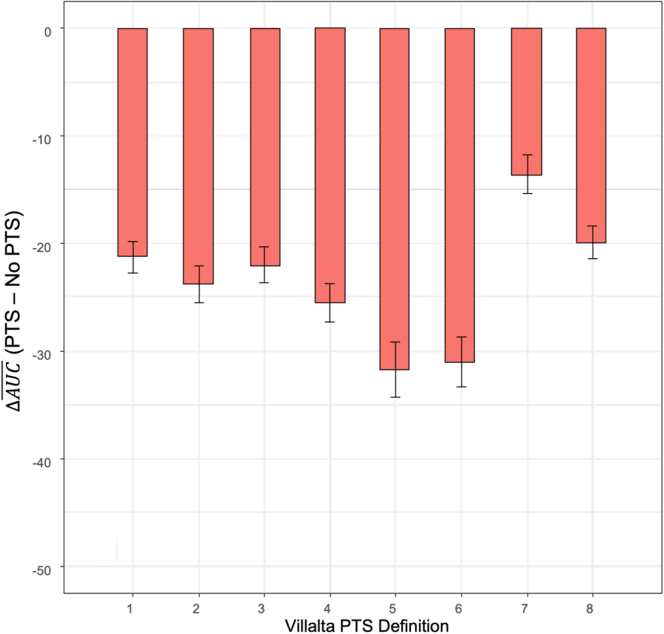
Differences of average fitted AUC ($\Delta \bar{AUC}$) in VEINES-QOL scores (PTS–No PTS) using 8 different approaches. Comparison between the fitted $\Delta \bar{AUC}$, calculated as the difference of the average AUC in VEINES-QOL scores between PTS and no PTS at 6-months and 24-months, for each PTS definition (1-8) as represented by the x-axis. The y-axis represents the difference of the average AUC between PTS and no PTS ($\Delta \bar{AUC}$). Error bars represent SE of mean. AUC, the difference of average area under the fitted curve between PTS and no PTS; PTS, postthrombotic syndrome; QOL, quality of life; VEINES, Venous Insufficiency Epidemiological and Economic Study

To compare the ability of the 8 approaches to discriminate between patients with poorer vs better QOL, the average fitted AUC of the VEINES-QOL scores obtained from the ATTRACT trial between 6 and 24 months were computed for patients with and without PTS, as defined by each of the 8 approaches, as shown in Table 3. A higher average AUC difference ($\Delta \bar{AUC}$) between PTS and no PTS indicates greater separation of VEINES-QOL scores between patients classified as having vs not having PTS. For the outcome of PTS, approaches 1, 2, and 3 had similar $\Delta \bar{AUC}$, mean (SD) of −21.2 (1.5), −23.7 (1.7), and −22.0 (1.6), respectively. For the outcome of moderate-to-severe PTS, approaches 5 and 6 had greater $\Delta \bar{AUC}$, mean (SD) −31.7 (2.6) and −31.0 (2.3), respectively, when compared with approach 4 with a $\Delta \bar{AUC}$ of −25.5 (1.8). However, the differences in $\Delta \bar{AUC}$ from approach 4 were not statistically significant. Finally, for the outcome of PTS, attempting to adjust the VS for contralateral CVI (approach 7) or restricting to the subset of patients without baseline contralateral CVI (approach 8) did not improve $\Delta \bar{AUC}$, mean (SD) of −13.6 (1.8) and −19.9 (1.5), respectively, compared with approach 1. These findings are also demonstrated in Figure 1, which depicts the difference in fitted $\Delta \bar{AUC}$ for each approach and correlate with the findings shown in Table 3. Approaches 1 to 3 demonstrate similar $\Delta \bar{AUC}$ between each other, whereas approaches 4 to 6 demonstrate higher $\Delta \bar{AUC}$ compared with approach 1. Finally, approach 7 shows a lower $\Delta \bar{AUC}$, and approach 8 a similar $\Delta \bar{AUC}$ when compared with approach 1.

To assess for statistically significant differences among the performance of the 8 approaches, the pair-wise differences in $\Delta \bar{AUC}$ are calculated and represented in Table 4. Approaches 1, 2, 3, 7, and 8 assess for “any PTS”. A negative average difference in $\Delta \bar{AUC}$ represents a greater performance of the PTS approach being compared, whereas a positive difference in $\Delta \bar{AUC}$ represents a worse performance of the PTS approach being compared. When comparing approaches 1 to 3, there were no statistically significant differences between PTS definitions, with differences in $\Delta \bar{AUC}$ (SE) between approaches 2 and 1, 3 and 1, and 3 and 2 of −2.5 (2.2; *P =*.27), −0.8 (2.2; *P =*.72), and 1.7 (2.3; *P =*.46), respectively. The difference in $\Delta \bar{AUC}$ (SE) between approach 7 and 1, 7 and 2, and 7 and 3 were 7.6 (2.3; *P =*.001), 10.1 (2.4; *P* <.001), and 8.4 (2.4; *P* <.001), respectively. Because the $\Delta \bar{AUC}$ for approach 7 was numerically and statistically significantly lower compared with approaches 1 to 3, this indicates that approaches 1 to 3 are better than approach 7 to discriminate between patients with poorer vs better QOL, as shown in Table 3. Finally, comparing approach 8 (ie, restricting to patients without baseline CVI) to approaches 1, 2, and 3 did not result in statistically significant differences, with $\Delta \bar{AUC}$ (SE) of 1.3 (2.1; *P =*.53), 3.8 (2.2; *P =*.085), and 2.1 (2.2; *P =*.33), respectively.

**Table 4:** Comparison of $\Delta \bar{AUC}$ for VEINES-QOL scores among different approaches to define any PTS and moderate-to-severe PTS.

Approaches being compared	Difference in $\Delta \bar{AUC}$ (SE)^a^	95% confidence interval^b^	*P* value^b^
Any PTS			
2-1^a^	−2.5 (2.2)	−6.9, 1.9	.27
3-1	−0.8 (2.2)	−5.1, 3.5	.72
3-2	1.7 (2.3)	−2.8, 6.2	.46
7-1	7.6 (2.3)	3.1, 12.1	.001
7-2	10.1 (2.4)	5.3, 14.9	<.001
7-3	8.4 (2.4)	3.7, 13.0	<.001
8-1	1.3 (2.1)	−2.8, 5.4	.53
8-2	3.8 (2.2)	−0.5, 8.2	.085
8-3	2.1 (2.2)	−2.1, 6.3	.33
8-7	−6.3 (2.3)	−10.8, −1.8	.001
Moderate-to-severe PTS			
5-4	−6.2 (3.1)	−12.3, −0.1	.046
6-4	−5.5 (2.9)	−11.1, 0.1	.053
6-5	0.7 (3.4)	−6.0, 7.4	.84
Additional comparisons			
4-1	−4.2 (2.3)	−8.8, 0.3	.067
5-1	−10.4 (3.0)	−16.3, −4.6	<.001
6-1	−9.8 (2.7)	−15.1, −4.5	<.001
4-2	−1.7 (2.4)	−6.5, 3.0	.47
5-2	−7.9 (3.1)	−13.9, −1.9	.001
6-2	−7.3 (2.8)	−12.8, −1.8	.001
4-3	−3.5 (2.4)	−8.1, 1.2	.15
5-3	−9.7 (3.0)	−15.6, −3.7	.001
6-3	−9.0 (2.8)	−14.4, −3.6	.001
7-4	11.8 (2.5)	7.0, 16.7	<.001
7-5	18.0 (3.1)	11.9, 24.1	<.001
7-6	17.4 (2.9)	11.8, 23.0	<.001
8-4	5.6 (2.3)	1.1, 10.0	.015
8-5	11.8 (2.9)	6.0, 17.5	<.001
8-6	11.1 (2.7)	5.8, 16.4	<.001

$\Delta \bar{AUC}$, the difference of average area under the fitted curve between PTS and no PTS; PTS, postthrombotic syndrome; SE, standard error.

a Differences in $\Delta \bar{AUC}$, e.g., $\Delta \bar{AUC}$ for approach 2 minus $\Delta \bar{AUC}$ for approach 1.

b Obtained by the bootstrap method. For all analyses, a *P* value of ≤.01 was considered to indicate statistical significance.

Approaches 4 to 6 assess for moderate-to-severe PTS. There was a numerical but not statistically significant difference between approaches 4 to 6, with differences in $\Delta \bar{AUC}$ (SE) between approach 4 and 5, 4 and 6, and 5 and 6 of −6.2 (3.1; *P =*.046), −5.5 (2.9; *P =*.053), and 0.7 (3.4; *P =*.84), respectively.

In terms of the correlation between VEINES-QOL scores and PTS severity, higher VS scores at baseline were associated with worse baseline VEINES-QOL scores, as expected, in a linear fashion until a VS score of ≥20, whereafter the data are sparse and show a plateau in VEINES-QOL scores (Supplementary Figure S1). Patients with mild PTS (VS ≤ 4), moderate PTS (5-9), and severe PTS (≥10) showed an overall improvement in VEINES-QOL score at 24-months when compared with baseline. The estimated difference in VEINES-QOL scores for patients with VS score of ≤4 vs patients with VS score of ≥5 remained relatively the same across the 24-month follow-up (Tables 1 and 2 in the Supplementary Appendix).

Finally, as would be expected, approaches that assessed for moderate-to-severe PTS tended to result in greater average $\Delta \bar{AUC}$ (SE) compared with approaches that assessed for “any PTS,” as shown in Table 4, owing to larger separation in VEINES-QOL scores in patients with more severe vs less severe or no PTS, compared with patients with “any PTS” vs no PTS.

## 4 Discussion

In this post hoc analysis of the ATTRACT trial database, we report 3 main findings. First, the study’s assessment of PTS using a single positive VS score of ≥5 performed as well as alternative methods of using the VS to evaluate for PTS. Second, the study’s assessment of moderate-to-severe PTS using a single positive VS score of ≥10 also performed as well as alternative methods of using VS to assess this outcome. Lastly, as expected, assessing for moderate-to-severe PTS resulted in a greater discrimination of VEINES-QOL score when compared with “any” PTS. From a clinical perspective, our results suggest that the current ISTH definition of PTS is reliable to be used in clinical trials.

The main objective of this exploratory analysis was to improve the ability to identify patients who develop clinically meaningful PTS after DVT and determine whether currently accepted PTS definitions can be reliably used in trials. To our knowledge, this is the first study that compared the different definitions of VS to determine whether one definition can more consistently discriminate between patients after DVT having poorer or better venous-specific disease QOL over follow-up than baseline. It has been suggested that the diagnosis of PTS based on one assessment of the VS would result in a less precise estimation of the true incidence of PTS compared with making a diagnosis of PTS based on ≥2 assessments [14]. In our analysis, we found no significant advantages of definitions that include >1 assessment compared with one assessment to define PTS.

In the Individualised versus Standard Duration of Elastic Compression Therapy for Prevention of Post-Thrombotic Syndrome (IDEAL DVT) study by ten Cate-Hoek et al. [26], the incidence of PTS was 51% in the individualized group and 45% in the standard group using the ISTH definition of PTS, whereas the incidence of PTS was 29% and 28% in the groups, respectively, using the Prandoni definition. The incidence of PTS in the IDEAL DVT study is concordant with our findings of 47% using approach 1 (ISTH definition), 23% using approach 2 (Prandoni definition), and 29% using approach 3. Similar to ten Cate-Hoek et al. [26], we found a numerical difference in the incidence of PTS in our study when using the ISTH vs Prandoni definitions. However, we showed that the definition used in the ATTRACT trial for “any” PTS (approach 1) is as reliable as approaches 2 and 3, suggesting that the ISTH definition may correctly estimate the incidence of PTS and may be preferable for greater convenience because it only requires one assessment of the VS score.

Although it is reported that the incidence of PTS may differ according to the definition used, it is thought that the incidence of moderate-to-severe PTS should be similar across the Prandoni and ISTH definitions because more severe PTS symptoms and signs are less likely to change over time [14]. We found in our analysis that using an approach that requires 2 assessments, whether consecutive or nonconsecutive, to define moderate-to-severe PTS did not lead to a significantly greater change in the AUC than the definition used in the ATTRACT trial (approach 4, a single VS score ≥10). Approaches 5 and 6 also yielded a smaller proportion of patients classified as having moderate-to-severe PTS (8% and 11% of the study sample, respectively) and were also found to have large standard errors, which could potentially falsely increase the change in $\Delta \bar{AUC}$. We showed that using the traditional ISTH definition (approach 4) for moderate-to-severe PTS is as reliable as approaches 5 (Prandoni definition of moderate-to-severe PTS) and 6. Hence, it appears that any single positive VS score of ≥10 assessed 6 months or later after DVT can be used to define moderate-to-severe PTS, which offers greater convenience.

Adjusting the VS score for baseline CVI, an approach based on the published results of the REVERSE study and SOX Trial, did not substantively improve the reliability of diagnosing clinically meaningful PTS. One of the reported limitations of the VS is the lack of specificity to venous disease [12,14,27]. A study by Galanaud et al. [17] comparing the VS in ipsilateral and contralateral legs after the first unprovoked DVT demonstrated that among patients with ipsilateral PTS (defined by a VS >4), 39.7% also had a VS score of >4 in the contralateral non-DVT affected leg, with similar distribution of symptoms and signs bilaterally. This suggested that close to half of the cases identified as PTS might be due to preexisting chronic venous disease. Galanaud et al. [17] suggested that assessing for contralateral VS over time in patients with an ipsilateral-VS score of ≥5 might allow for better selection of patients with “true” PTS for participation in PTS management trials. The specificity to detect “true” PTS thus may rely, at least in part, on the presence or absence of preexisting CVI. Our study showed that using the traditional ISTH definition (approach 1) for PTS is as reliable as an approach adjusting for baseline CVI, and specific enough to define PTS without the need to account for preexisting (ie, contralateral) venous disease. Therefore, the former approach is preferred for greater convenience. These findings are in keeping with additional work by Galanaud et al., [29] in which the contralateral VS was only mildly correlated to ipsilateral-VS, and the proportion of PTS attributable to CVI was modest and lower than previously reported [28].

The VS score was obtained in 83% (576/691) of patients at 6 months and in 72% (498/691) at 24-month follow-up. Among the enrolled 691 patients, 40% (276) of patients missed at least one PTS visit between 6- and 24-month follow-up. Moreover, 12% (80) of patients missed all 4 PTS visits between 6- and 24-month follow-up and were classified as not having PTS, which likely underestimated the overall number of patients defined as having PTS, regardless of the definition used.

Our study has several strengths. This subanalysis was performed on a large, multicenter, assessor-blinded, prospectively recruited randomized clinical trial. The overall rate of PTS in the ATTRACT trial is consistent with previously reported rates in other PTS trials [12,30]. The definitions of PTS were based on previous trials and ISTH standards. We used the VEINES-QOL instrument, a well validated and reliable instrument that is more sensitive than generic measures in detecting treatment effects and changes over time, to measure disease-specific QOL and correlate it with the VS score to define clinically meaningful PTS [6,12,18]. Although there exists no reference standard measure for PTS, QOL is the best available metric for patient-important PTS, because PTS impacts patient well-being, and QOL scores have been previously shown to correlate with the VS score.

We systemically collected data on baseline patient characteristics that could independently affect QOL, such as body mass index and age, such that these variables could be controlled for in our growth curve model. We decided to use the AUC as this enabled equivalent weighting of all time points.

Our study has several limitations. The performance of the 8 approaches used to define PTS was only tested in the ATTRACT trial population and has not been evaluated in other populations of patients with DVT. Our study population was limited to patients with symptomatic, proximal DVT involving the femoral, common femoral, or iliac veins and did not include patients with isolated distal DVT, which may limit the generalizability of our results. Because of small numbers of patients in some subgroups, we were unable to compare differences in performance of the various definitions of PTS in patients with femoral, common femoral, and iliac vein DVT.

One limitation of the ATTRACT trial and this sub analysis is the substantial number of missing PTS assessments. Although most patients had PTS assessments at multiple study visits within 24 months follow-up, approximately 12% of patients missed all 4 visits and 30% of patients did not have a complete VEINES-QOL score assessment at 24 months. Although this is unlikely to have impacted the sensitivity in discriminating the best approach to defining PTS, the missing PTS assessments may have underestimated the number of patients defined as having PTS.

We report in this sub analysis the comparison between definitions of “any” PTS to no PTS, and moderate-to-severe PTS to no/mild PTS, outcomes that were used in the ATTRACT trial. However, further categorization of PTS as none, mild, moderate, and severe may have practical value that has not been addressed in this study.

Our analysis only looked at VS scores in relation to venous disease-specific QOL (VEINES-QOL) scores and not generic QOL scores over follow-up, but this was felt to be appropriate as VEINES-QOL is a venous disease-specific measure. The sample size of some of the subgroups used for each definition were small, with an associated elevated SE when calculating for the difference in AUC. This means that there may be a falsely elevated change in AUC that may not be of clinical significance.

## 5 Conclusion

We conclude that the ISTH definition of PTS, as denoted by a VS score of ≥5 in the ipsilateral leg on a single assessment, as used in the ATTRACT trial, is a reliable definition for PTS. Repeating the assessment or accounting for the contralateral leg VS score does not improve upon the original assessment. We suggest that a VS score of ≥10 on a single assessment can be used to define moderate-to-severe PTS with similar accuracy to 2 consecutive or nonconsecutive positive assessments and is preferred due to greater convenience.
