# Supplementary material for: Exploring the Villalta scale to capture postthrombotic syndrome using alternative approaches: A subanalysis of the ATTRACT trial
Source: Res Pract Thromb Haemost. 2022 Dec 27;7(1):100032. doi: 10.1016/j.rpth.2022.100032 (PMC10031339; doi:10.1016/j.rpth.2022.100032)
Supplement: Appendix [file mmc1.docx]

**Appendix 1**

Table 1: QOL by Villalta Score (≤ 4 vs ≥ 5) from baseline to 24-months follow-up

| **Outcome Measure** | **Villalta Score ≤ 4** | | **Villalta Score ≥ 5** | | **Difference** | |
| --- | --- | --- | --- | --- | --- | --- |
|  | **n** | **mean (SD)** | **n** | **mean (SD)** | **Estimate (SD)** | **P-value^¶^** |
| **VEINES-QOL:** |  |  |  |  |  |  |
| At baseline | 121 | 69.00 (20.42) | 555 | 46.89 (22.60) | 22.10 (22.23) | <0.001 |
| At 30 days | 356 | 74.34 (19.22) | 279 | 48.02 (22.79) | 26.31 (20.87) | <0.001 |
| At 6 months | 382 | 84.77 (15.05) | 190 | 55.54 (25.03) | 29.23 (18.95) | <0.001 |
| At 12 months | 347 | 88.02 (13.74) | 179 | 57.80 (24.35) | 30.22 (18.06) | <0.001 |
| At 18 months | 306 | 89.29 (13.64) | 161 | 59.01 (23.21) | 30.27 (17.53) | <0.001 |
| At 24 months | 322 | 88.71 (13.00) | 158 | 59.72 (23.02) | 28.99 (16.96 | <0.001 |

VEINES-QOL, Venous Insufficiency Epidemiological and Economic Study - Quality of Life/Symptoms; SD, standard deviation.

¶ Obtained by the bootstrap method. For all analyses, a P-value ≤ 0.01 was considered to indicate statistical significance.

Table 2: QOL by Villalta Score severity from baseline to 24-months follow-up

| **Outcome Measure** | **Villalta Score ≤ 4** | | **Villalta Score 5-9** | | **Villalta Score 10-14** | | **Villalta Score ≥ 15** | | **P-value^¶^** |
| --- | --- | --- | --- | --- | --- | --- | --- | --- | --- |
|  | **n** | **mean (SD)** | **n** | **mean (SD)** | **n** | **mean (SD)** | **n** | **mean (SD)** |  |
| **VEINES-QOL:** |  |  |  |  |  |  |  |  |  |
| At baseline | 121 | 69.00 (20.42) | 235 | 55.95 (22.12) | 190 | 43.34 (19.97) | 130 | 35.70 (20.70) | <0.001 |
| At 30 days | 356 | 74.34 (19.22) | 186 | 53.25 (21.28) | 55 | 41.64 (22.25) | 38 | 31.69 (21.09) | <0.001 |
| At 6 months | 382 | 84.77 (15.05) | 119 | 63.13 (22.87) | 38 | 47.00 (20.79) | 33 | 37.97 (25.56) | <0.001 |
| At 12 months | 347 | 88.02 (13.74) | 116 | 67.43 (20.09) | 45 | 42.27 (19.17) | 18 | 34.55 (26.17) | <0.001 |
| At 18 months | 306 | 89.29 (13.64) | 98 | 66.21 (20.47) | 40 | 45.33 (19.90) | 23 | 52.14 (27.33) | <0.001 |
| At 24 months | 322 | 88.71 (13.00) | 94 | 67.07 (22.00) | 38 | 51.02 (17.15) | 26 | 45.87 (23.99) | <0.001 |

VEINES-QOL, Venous Insufficiency Epidemiological and Economic Study - Quality of Life/Symptoms; SD, standard deviation.

¶ Obtained by the bootstrap method. For all analyses, a P-value ≤ 0.01 was considered to indicate statistical significance.

Figure 1: Scatterplot of VEINES-QOL scores by Villalta Score at baseline and 12-months follow-up


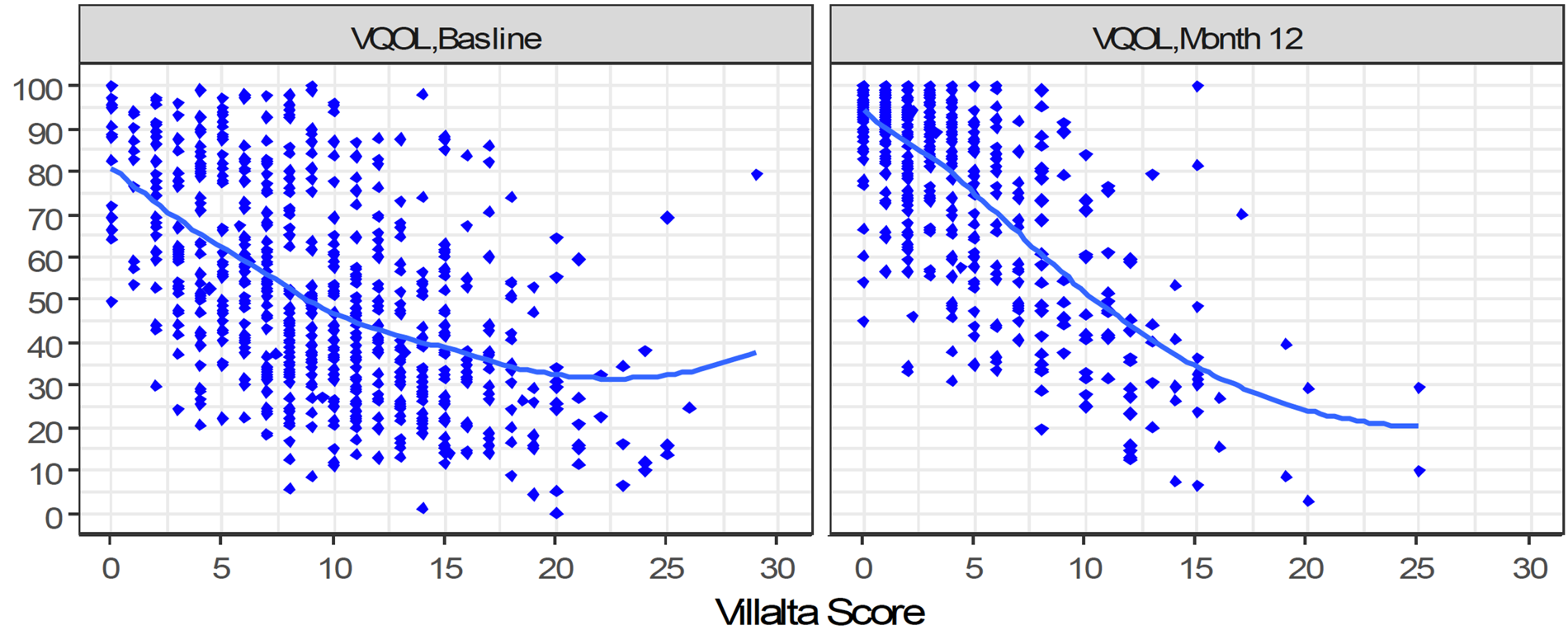


Scatterplot of the individual VEINES-QOL scores by Villalta Scores at baseline (left) and at 12-months follow-up (right). The y-axis represents the VEINES-QOL score on a scale from 0 to 100 points. The x-axis represents the Villalta Score from 0-30 points. The blue line represents the mean VEINES-QOL score as a function of the VS score.
